# Use of Machine Learning in Predicting the Risk of Cirrhosis in Autoimmune Hepatitis Based on Clinical and Immunological Indicators

**DOI:** 10.3390/diagnostics16070974

**Published:** 2026-03-25

**Authors:** Nazugum Ashimova, Aigul Raissova, Elmira Kuantay, Moldir Khozhakhmedova, Nurgul Aldabergenova, Madina Suleimenova, Kuat Abzaliyev, Nassyrova Nargiza, Ruslan Kulmanbetov, Alexander Nersesov

**Affiliations:** 1 Department of Gastroenterology, Asfendiyarov Kazakh National Medical University, Almaty 050012, Kazakhstan; nazugumashimova90@gmail.com (N.A.); ram-79@mail.ru (A.R.); kuantaielmira08@gmail.com (E.K.); alexander.nersesov@gmail.com (A.N.); 2 Institute of Gastroenterology, Hepatology and Metabolism “Interna Clinic”, Almaty 050000, Kazakhstan; moldir.khozhakhmedova@gmail.com (M.K.); n.aldabergenova@inbox.ru (N.A.); 3 AI and Big Data Department, Faculty of Information Technology, Al-Farabi Kazakh National University, Almaty 050040, Kazakhstan; kuatabzaliev1961@gmail.com; 4 Department of Research, Kazakhstan-Russian Medical University, Almaty 050004, Kazakhstan; nassyrova.n94@gmail.com; 5 Department of Orthodontics, Asfendiyarov Kazakh National Medical University, Almaty 050012, Kazakhstan

**Keywords:** autoimmune hepatitis, cirrhosis, overlap syndrome, machine learning, predictive model

## Abstract

**Background/Objectives**: Autoimmune hepatitis (AIH) is a chronic immune-mediated inflammatory liver disease that, if not diagnosed and treated promptly, leads to cirrhosis and liver failure. Data on AIH in Central Asia, including Kazakhstan, remain limited. The aim of this study was to characterize the clinical profile of AIH in a Kazakhstani patient cohort, determine the timeliness of diagnosis, and develop an interpretable machine learning model for detecting liver fibrosis based on routine clinical and laboratory parameters. **Methods**: A retrospective observational study of adult patients with a diagnosis of AIH between 2015 and 2025 was conducted. Demographic, laboratory, instrumental, and histological data of patients with AIH were extracted from medical records. All statistical analyses were performed using SPSS 22.0. **Results**: The study included 240 patients with a mean age of 49.3 ± 14.3 years; 87.1% of patients were women. The Random Forest model showed the best results: ROC-AUC of 0.803 ± 0.057, PR-AUC of 0.868 ± 0.044, Brier of 0.180 ± 0.017, sensitivity of 0.816, and specificity of 0.641. SHAP analysis confirmed that platelet count, age, INR, disease duration, and bilirubin and albumin levels made the greatest contribution to the prognosis. **Conclusions**: This retrospective observational study of AIH in Kazakhstan identified a patient population characterized by late diagnosis and advanced disease stages at presentation, a high frequency of overlapping autoimmune liver diseases, and a significant burden of metabolic and extrahepatic autoimmune comorbidities. The results demonstrate that an interpretable machine learning model based on routine biomarkers can effectively detect fibrosis and provide clinically interpretable risk factors.

## 1. Introduction

Autoimmune hepatitis (AIH) is an immune-mediated inflammatory liver disease of unknown etiology [1]. It is characterized by chronic necroinflammatory changes in the liver, the presence of circulating autoantibodies, and elevated immunoglobulin G (IgG) levels [2]. AIH can affect both children and adults worldwide; according to the literature, the incidence is approximately 0.7–2.0 per 100,000 population per year, and the prevalence ranges from 4 to 25 per 100,000 in different populations [3,4]. The disease exhibits a marked female predominance: approximately 75–80% of patients with AIH are women [5]. This gender predisposition is observed in many ethnic groups and regions and is likely associated with immunogenetic and hormonal factors [6]. The clinical manifestations of AIH vary widely, from asymptomatic elevation of liver enzymes to chronic hepatitis, which may progress undetected to cirrhosis or even hepatocellular carcinoma, and in rare cases to acute severe hepatitis or fulminant liver failure [7].

Delayed recognition of AIH can have serious consequences. Untreated AIH is associated with a high risk of progression to cirrhosis and liver-related mortality, while timely immunosuppressive therapy significantly improves prognosis [8,9]. However, late diagnosis remains a problem worldwide. Studies in South Asia and other regions have shown that many cases of AIH are diagnosed at the cirrhosis stage [4,10,11,12,13]. Reasons for diagnostic delay include insufficient physician suspicion of AIH, misdiagnosis of other liver diseases, and seronegative forms that do not meet standard diagnostic criteria. Even in well-studied cohorts, up to one-third of patients with AIH may lack classical autoantibodies; such patients typically have a more aggressive course with rapid progression to cirrhosis [14,15]. Furthermore, AIH is frequently associated with other autoimmune liver diseases. Overlap with primary biliary cholangitis (PBC) or primary sclerosing cholangitis (PSC) is observed in approximately 5–10% of cases [16,17], further complicating diagnosis and treatment.

Artificial intelligence tools are increasingly used in medical research to address specific problems. Machine learning (ML) algorithms can validate existing methods and support new clinical decisions [18]. Various methods have been used for the early diagnosis and prognosis of liver diseases. In a study by Alenizi and Al-Karawi, several machine learning models were evaluated for hepatitis detection using classification metrics such as accuracy, precision, and recall. Among them, the Random Forest algorithm showed the best performance, with an accuracy of 87.76% [19]. To predict the presence of liver diseases based on clinical and biochemical data with high accuracy, other researchers used MICE imputation, PCA for dimensionality reduction, and compared ANN, SVM, and RandomForest, finding that RandomForest provided the best accuracy of ~98.14% [20]. A paper describes a machine learning model for predicting the risk of hepatocellular carcinoma (HCC) in patients with metabolic-associated fatty liver disease (MASLD) based on standard clinical and laboratory data from the EHR; the model was trained on the UC Davis cohort and validated on an independent UC San Francisco cohort. Validation showed an AUC of ≈0.97 and an accuracy of ~92%, with FIB-4 (a noninvasive fibrosis index) being the strongest single predictor [21]. Thus, this suggests that ML methods predict liver diseases based on risk factors, which may improve patient diagnosis based on the obtained results.

Data on AIH in Central Asia, including Kazakhstan, are extremely limited, and disease characteristics in the Kazakh population have been virtually unknown. Understanding the clinical presentation and outcomes of AIH in this region is crucial for improving diagnosis and patient management. We hypothesized that, due to a lack of awareness, patients in Kazakhstan may experience significant diagnostic delays and are more likely to have advanced disease (fibrosis/cirrhosis) at diagnosis compared to Western cohorts.

The aim of this study was to characterize the clinical profile of AIH in a Kazakhstani patient cohort, determine the timeliness of diagnosis, and develop an interpretable machine learning model for detecting liver fibrosis based on routine clinical and laboratory parameters. Ultimately, the study aims to identify areas that can facilitate earlier diagnosis and improve outcomes for patients with AIH in this region.

## 2. Materials and Methods

This retrospective study of 240 adult patients with a probable or definite diagnosis of AIH was conducted at the Institute of Gastroenterology, Hepatology, and Metabolism in Almaty, Republic of Kazakhstan. The diagnosis of AIH was established based on the International Autoimmune Hepatitis Group criteria, taking into account the corresponding histology, an increase in serum IgG, the presence of specific autoantibodies (ANA, SMA, LKM, SLA), and the absence of viral hepatitis. AIH-PBC was established according to the Paris criteria (≥2 of 3 AMA criteria—positivity, ALP ≥ 2 ULN/GGT ≥ 5 ULN, histological signs of destructive cholangitis). AIH-PSC was diagnosed based on the presence of diagnostic features of AIH in combination with characteristic cholangiography changes in the PSC according to MRCP or histological changes in the PSC of small ducts.

The inclusion criteria were age 18 years and older and the presence of autoimmune hepatitis.

The exclusion criteria were the presence of viral hepatitis, significant alcohol consumption (more than 30 g/day for men and 20 g/day for women), other causes of chronic liver disease, hepatocellular carcinoma, pregnancy, and failure to meet the inclusion criteria.

The study was conducted in accordance with the Declaration of Helsinki and approved by the Institutional Review Board of Asfendiyarov Kazakh National Medical University (protocol code 163; date of approval: 29 April 2025).

Demographic, laboratory, instrumental and histological data were obtained from medical records.

Immunological testing included determination of antinuclear antibodies (ANA); if negative, antibodies to smooth muscle (ASMA), antibodies to liver and kidney microsomes (anti-LKM), and antibodies to soluble liver antigen/liver-pancreatic antigen (anti-SLA/LP) were determined. Autoantibodies were determined using indirect immunofluorescence; a titer of ≥1:80 was considered positive.

All patients underwent transient elastography (TE) (LSM), and ARFI and Fib-4 were calculated. TE was performed using a FibroScan Expert 630 (Echosens, Paris, France) by an experienced specialist who has performed over 400 successful studies. Liver stiffness scores (LSM) were interpreted based on validated AIH-specific cutoff values, where ≥9 kPa corresponds to significant fibrosis (≥F3) and 12.5–16 kPa to cirrhosis (F4). The study used TE cut-off values specific for AIH (≥9 kPa ≥ F3, 12.5–16 kPa—F4); however, the cohort included patients with both isolated AIH and overlap syndromes and MASLD, while the cut-off values used are close to the ranges used in chronic liver diseases, where cut-off values of 8–12 kPa and 12–15 kPa indicate severe fibrosis and compensated advanced liver disease (cACLD). Using an A-mode ultrasound image obtained from a FibroScan device, a liver parenchyma area of at least 6 cm in thickness was identified, after which stiffness was measured in 10 kPa. Standard quality criteria were met: ≥10 valid measurements, ≥60% success rate, and IQR ≤ 30%. The APRI index was calculated in parallel based on AST and platelet indices and classified as no fibrosis (≤0.5) or significant fibrosis (≥1.5).

### Statistical Analysis

Statistical analysis of the data was performed using SPSS 22.0. Descriptive statistics were used to analyze demographic data and determine the mean, median, standard deviation, and range. Diagnostic delay was defined as the interval from the onset of first symptoms and elevated liver enzymes to diagnosis and was classified as >12 months or ≤12 months.

When developing the interpretable machine learning model, the target was defined as advanced fibrosis/cirrhosis (F3–F4) based on transient elastography and served as a binary outcome (F3–F4: 166/240, 69.2%; F0–F2: 74/240, 30.8%). Liver biopsy data were only available for a subset of patients and were used for descriptive analysis but were not included in the definition of the target variable in the model. Predictors included demographics (age, gender, region), lifestyle factors (alcohol, smoking), medical history (disease duration), and routine laboratory biomarkers reflecting liver function and systemic changes (e.g., platelets, bilirubin, albumin, INR, transaminases, hemoglobin, inflammatory markers). Multiple-choice variables were converted into binary indicators (multi-hot coding), and additional summary features (“any” and “count”) were derived. All variables were converted to numeric format. Missing values were handled using median imputation with parameters fitted on training subsets only to prevent information leakage. Two models were trained and compared: ElasticNet logistic regression (with feature standardization) and Random Forest (no scaling required). Model performance was evaluated using a stratified nested cross-validation framework (5 outer folds/3 inner folds) with hyperparameter tuning via randomized search optimizing ROC-AUC. Class imbalance was handled using class_weight = ‘balanced’ and no resampling (SMOTE/over-/under-sampling) to preserve prevalence and probabilistic interpretability. Performance was assessed using ROC-AUC, PR-AUC, and Brier score. Beyond the reference threshold of 0.5, we also evaluated clinically motivated thresholds (Youden-optimal and a screening-oriented sensitivity ≥ 0.85 operating point), with thresholds selected within each outer-fold training set using inner-CV out-of-fold probabilities and applied to the held-out outer test fold. Predictor contributions were interpreted using permutation importance and SHAP. Decision curve analysis (DCA) was performed to assess clinical utility by estimating net benefit across a range of threshold probabilities. Net benefit was calculated as NB = TP/N − FP/N × (pt/(1 − pt)) and compared with “treat-all” and “treat-none” strategies. To prevent optimistic bias, DCA was computed using out-of-sample probabilities from the outer test folds of the nested cross-validation.

## 3. Results

### 3.1. Patient Characteristics

A total of 240 patients with autoimmune hepatitis (AIH) were included (Table 1). The mean age at diagnosis was 49.3 ± 14.3 years (median 51; IQR 39–61), and 209 (87.1%) were female. The mean BMI was 25.1 ± 4.5 kg/m^2^; 117 (48.8%) had overweight or obesity. The median disease duration prior to diagnosis was 24 months (IQR 12–48; mean 36.3 ± 44.8 months). Jaundice was found in 52 (21.7%) and pruritus in 87 (36.2%).

Table 2 demonstrates that the majority of patients showed typical autoimmune serological signs; positive ANA was noted in 85.8%, elevated IgG levels in 76.7%, and elevated γ-globulins in 71.2%, as well as a high prevalence of positive AMA (45.8%), including AMA-M2 (17.5%).

### 3.2. Fibrosis Assessment

**Figure 1:**
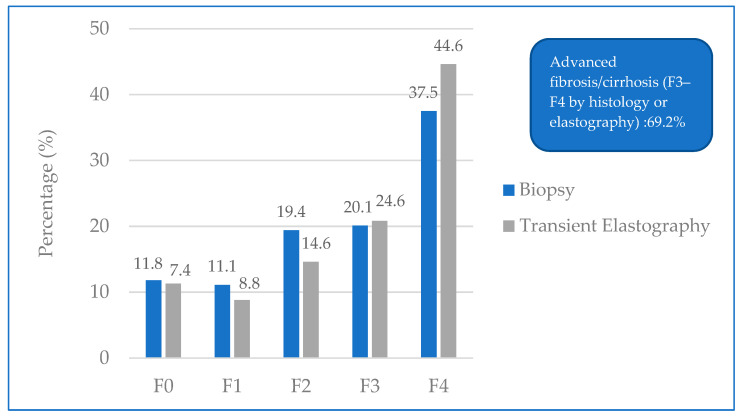
Fibrosis stages according to biopsy (*n* = 144) and transient elastography (*n* = 240).

Liver biopsy was performed in 144 patients, of whom 57.6% had advanced fibrosis (F3–F4) on histological examination. Elastography was performed in all patients, and 69.2% showed F3–F4 fibrosis (Figure 1).

### 3.3. Concomitant Diseases

**Figure 2:**
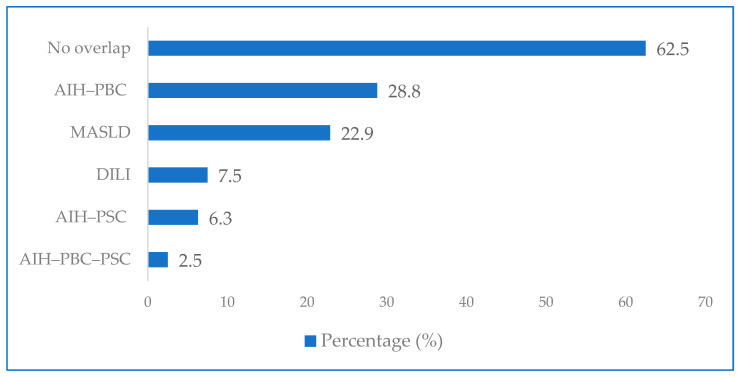
Concomitant liver diseases in patients with AIH.

The distribution of concomitant liver diseases in patients with AIH was as follows: isolated autoimmune hepatitis was detected in 62.5% of patients, while AIH-PBC overlap syndrome was observed in 28.8%, AIH-PSC overlap syndrome in 6.3%, and AIH-PBC-PSC triple overlap syndrome in 2.5%. MASLD was detected in 22.9% of patients, and a history of drug-induced liver injury (DILI) was recorded in 7.5% (Figure 2).

**Figure 3:**
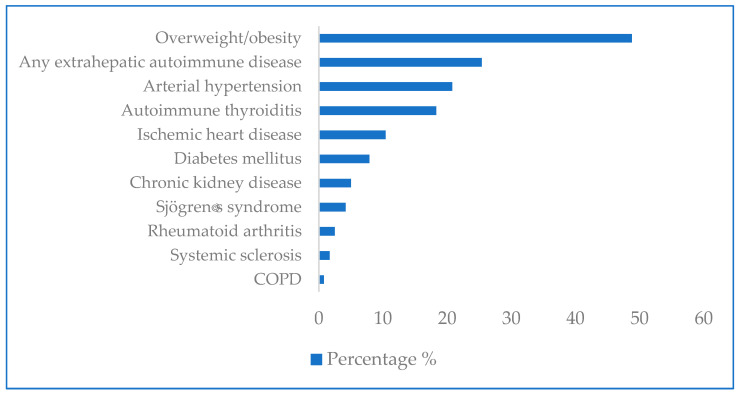
Concomitant diseases in patients with AIH.

Extrahepatic autoimmune diseases were diagnosed in 25.4% of patients, including autoimmune thyroiditis (18.3%), Sjogren’s syndrome (4.2%), rheumatoid arthritis (2.5%), and systemic sclerosis (1.7%). The most common comorbid pathology was overweight and obesity, observed in 48.8% of patients. Arterial hypertension (AH) was detected in 20.8%, coronary heart disease (CHD) in 10.4%, diabetes mellitus (DM) in 7.9%, and chronic kidney disease (CKD) in 5% (Figure 3).

### 3.4. Diagnostic Delay

In the multivariate logistic regression analysis (Table 3), a statistically significant association was found between a diagnostic delay of more than 12 months and the detection of severe and advanced fibrosis (≥F3). Advanced fibrosis (≥F3) was statistically significantly associated with a diagnostic delay of more than 12 months (OR 2.06; 95% CI 1.15–3.71; *p* = 0.0158). Age (OR 0.99; 95% CI 0.97–1.01; *p* = 0.4719) and female gender (OR 0.60; 95% CI 0.27–1.37; *p* = 0.2255) were not statistically significant predictors of diagnostic delay.

### 3.5. An Interpretable Machine Learning Model for Predicting Advanced Fibrosis

**Figure 4:**
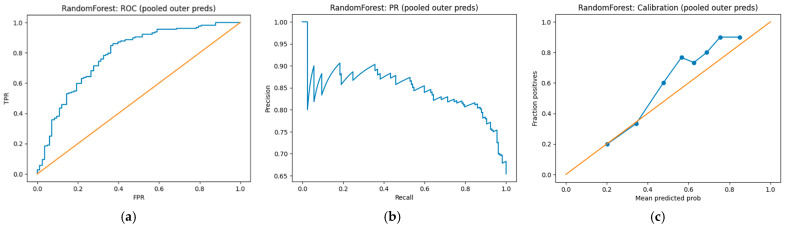
The overall results of external cross-validation: (**a**) ROC curve (pooled outer-fold predictions); (**b**) precision–recall curve; (**c**) calibration curve.

To build a predictive model, the target feature was a binary problem for detecting advanced fibrosis: F3–F4 (class 1) versus F0–F2 (class 0). The quality of the models was assessed using nested cross-validation (5 external folds and 3 internal folds for hyperparameter selection), which ensures an unbiased comparison of algorithms with simultaneous optimization. Two models were compared: ElasticNet logistic regression (ElasticNet-LR) and Random Forest. According to the overall external cross-validation results, the Random Forest model was the best model in discrimination, with ROC-AUC = 0.803 ± 0.057, PR-AUC = 0.868 ± 0.044, and Brier score = 0.180 ± 0.017 (Figure 4). At a probability threshold of 0.5, Random Forest demonstrated a sensitivity of 0.816 and a specificity of 0.641, indicating a good balance between identifying patients with F3–F4 and correctly classifying patients with F0–F2. ElasticNet-LR also showed competitive results (ROC-AUC = 0.769 ± 0.077, PR-AUC = 0.851 ± 0.059, Brier score = 0.177 ± 0.017), with the model having higher sensitivity (0.905) but significantly lower specificity (0.485); that is, it more often classified patients into the advanced fibrosis group, which is typical for linear models with class imbalance and the chosen fixed threshold.

To enhance clinical interpretability, threshold-dependent performance was evaluated in addition to the reference threshold of 0.5. At threshold = 0.5, RandomForest achieved sensitivity = 0.848 ± 0.045 and specificity = 0.557 ± 0.184. The Youden-optimal threshold was 0.591 ± 0.044, yielding sensitivity = 0.766 ± 0.153 and specificity = 0.701 ± 0.122, indicating improved specificity at the expense of sensitivity. For a screening-oriented operating point targeting sensitivity ≥ 0.85, the selected threshold was 0.474 ± 0.030, resulting in sensitivity = 0.867 ± 0.033 and specificity = 0.533 ± 0.165. Detailed threshold-dependent metrics (including PPV/NPV) are reported in Table 4.

**Figure 5:**
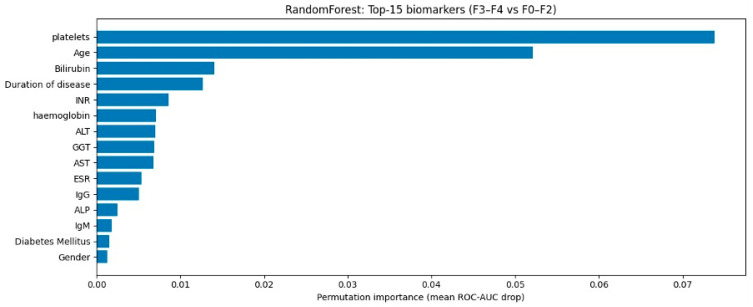
Feature importance (permutation importance of top 15 biomarkers).

A permutation importance analysis revealed that laboratory markers and clinical and anamnestic characteristics reflecting the progression of chronic liver disease and its systemic consequences contributed most to predicting advanced fibrosis (F3–F4). According to permutation importance, the most informative predictors for the best model (Random Forest) were platelet count, age, INR/INR, and disease duration, as well as liver function-related parameters (e.g., bilirubin and albumin) and signs of inflammation/comorbid conditions (Figure 5).

**Figure 6:**
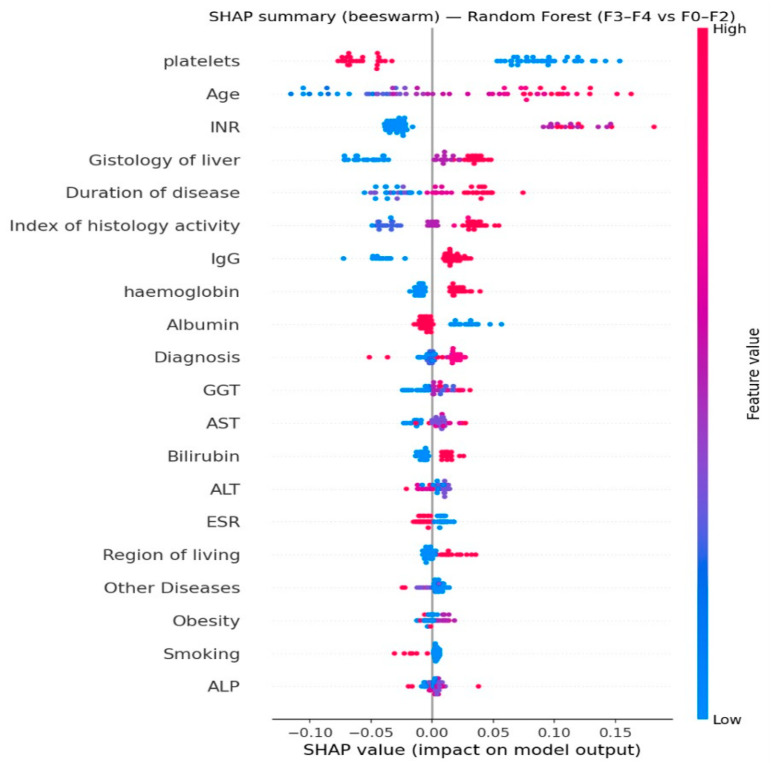
SHAP summary (beeswarm) plot.

SHAP analysis (for Random Forest) confirmed the dominant role of these features and allowed for the assessment of the direction of their influence on the probability of classifying a patient into the F3–F4 group (Figure 6).

**Figure 7:**
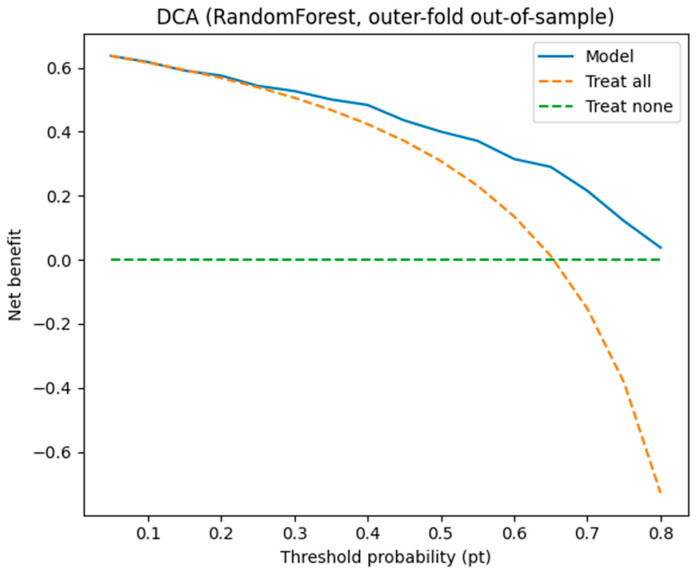
DCA for RandomForest.

Decision curve analysis (DCA) was performed using out-of-sample probabilities from the outer test folds (*n* = 240; prevalence = 0.654) to quantify clinical utility. The Random Forest model provided higher net benefit than both the “treat-all” and “treat-none” strategies across a wide range of clinically relevant threshold probabilities (approximately higher pt of 0.05–0.80), indicating potential usefulness for triage and risk-guided decision-making. For example, the model’s net benefits were 0.6176, 0.5750, 0.5268, and 0.4833 at pt = 0.10, 0.20, 0.30, and 0.40, respectively (Figure 7).

## 4. Discussion

### 4.1. Main Findings and Explanations

To our knowledge, this is the first study that aimed to characterize the clinical profile of patients with AIH and develop an interpretable machine learning model for predicting advanced liver fibrosis (F3–F4) based on routine clinical and laboratory parameters in a Kazakhstani cohort. The results demonstrate both expected similarities to global cohorts of patients with AIH and a number of differences that likely reflect regional characteristics and challenges in timely disease recognition.

One of the most significant and concerning findings was that most patients already had advanced liver fibrosis or cirrhosis at diagnosis. In other words, a significant proportion of patients were diagnosed at a late stage of the disease, often with clinical or histological evidence of cirrhosis. This is a matter of serious concern and exceeds the rates reported in a number of other regions—approximately one-third of adult patients with AIH are diagnosed with cirrhosis [22]. In our cohort, approximately two-thirds of patients had advanced fibrosis, indicating either an even more significant diagnostic delay or a more aggressive course of the disease.

AIH can be asymptomatic or have non-specific manifestations: it is estimated that approximately 25–34% of patients are initially asymptomatic [23]. The time interval from disease onset to clinical manifestation can vary from 2 months to 10 years (with a mean of approximately 32 months) [22,24], which is consistent with our finding of a median diagnostic delay of 24 months. During this period of subclinical or unrecognized disease, ongoing immune-mediated hepatocyte injury may lead to fibrosis progression. In our cohort, advanced fibrosis was statistically significantly associated with diagnostic delay, indicating that patients with more advanced liver disease were more likely to have a late diagnosis. These data highlight an important point repeatedly noted in the literature: if not promptly recognized and treated, AIH can insidiously progress to cirrhosis, which is associated with a poor prognosis [25]. Historically, untreated AIH has been associated with high mortality over several years, whereas timely immunosuppressive therapy provides excellent long-term survival [26]. Thus, the advanced stage of the disease in a significant proportion of our patients likely reflects missed opportunities for earlier diagnosis.

Another key finding of our study is the high frequency of overlap syndromes, particularly the combination of AIH and PBC. Almost one in three patients in our cohort showed signs of AIH-PBC overlap. This rate is significantly higher than in most Western cohorts, where the prevalence of such overlap is typically around 5–10% [27,28]. It should be noted that the frequency of overlap syndromes varies significantly (from approximately 2% to 20%) depending on the diagnostic criteria used [29]. It is also possible that genetic or environmental factors in Kazakhstan predispose to the development of overlapping autoimmune liver diseases. The high frequency of overlap forms may partially explain the widespread advanced fibrosis in our sample.

Typical serological features of AIH were observed, such as a high frequency of antinuclear antibodies (ANA) and elevated IgG levels. However, the prevalence of antimitochondrial antibodies (AMA) was unusually high, indicating a significant number of cases with overlap between AIH and primary biliary cholangitis (PBC). From a serological perspective, the high proportion of AMA-positive patients (approximately 46%) deserves special attention. Although low-titer AMA can sometimes be detected in “pure” AIH, such a high prevalence of these antibodies is a strong indicator for the presence of concomitant PBC or an overlap syndrome.

Our study also showed that 25% of patients had at least one extrahepatic autoimmune disease (including autoimmune thyroiditis, Sjögren’s syndrome, rheumatoid arthritis, or systemic sclerosis). These data are consistent with previously published results, according to which 20–40% of patients with AIH are found to have other autoimmune diseases [3,30]. The most common comorbidity in our cohort was autoimmune thyroiditis, which is consistent with the literature indicating a high frequency of thyroid pathology in patients with AIH [31].

Metabolic comorbidities were also a prominent feature of our cohort, which may impact disease severity and treatment approaches. Nearly half of patients were overweight or obese (BMI ≥ 25 kg/m^2^), and approximately 23% were diagnosed with MASLD, which is consistent with international data [32].

Another important observation was the long duration of symptoms before diagnosis—the median diagnostic delay was approximately two years—indicating the difficulty of early detection of AIH in this setting.

### 4.2. Machine Learning Provision

Machine learning applied to the development of a prognostic model demonstrated that a combination of routine clinical and laboratory parameters enables the accurate detection of advanced fibrosis (F3–F4) relative to F0–F2. Model quality was assessed using nested cross-validation (5 external folds and 3 internal folds for hyperparameter selection), which ensures an unbiased comparison of algorithms during simultaneous optimization. An ROC-AUC value of approximately 0.80 for Random Forest indicates reliable discrimination and the presence of a stable signal in the data. Importantly, nested cross-validation was used for the evaluation, which reduces the risk of metric overestimation compared to a simple train/test scheme or non-nested cross-validation, particularly during hyperparameter selection. From a practical perspective, Random Forest provides a better balance of sensitivity and specificity compared to ElasticNet-LR. This could be crucial for clinical application: when screening for advanced fibrosis, high sensitivity is typically preferred to minimize missing high-risk patients, but excessively lower specificity leads to an increase in false positives and routing overload. In our study, ElasticNet-LR demonstrated high sensitivity but low specificity, potentially making it less suitable as a standalone decision-making tool at a fixed threshold of 0.5. However, the linear model retains the advantage of interpretability and can be used as a transparent alternative during threshold optimization or calibration.

The identified key features have a clinically plausible interpretation. A decrease in platelets is a known indirect marker of portal hypertension and fibrosis progression. An increase in INR/INR and a decrease in albumin reflect a deterioration in the liver’s synthetic function, which is more often observed in advanced stages. An increase in bilirubin is associated with impaired excretory function and possible cholestasis. The significance of age and disease duration corresponds to the accumulation of damage and the duration of exposure to etiologic factors. Thus, the model relies not on random correlations, but on a biologically and clinically sound progression profile.

Probability calibration deserves special attention. Although Random Forest’s Brier score is acceptable, calibration curves may underestimate or overestimate risk in certain probability ranges. For the practical use of probabilities (e.g., as a risk scale), it is advisable to additionally consider post-calibration (isotonic regression or Platt scaling) and evaluate calibration on an external sample. It is also recommended to optimize the probability threshold for the clinical purpose (e.g., to ensure sensitivity ≥ 0.85 during screening) and additionally present the confusion matrix and PPV/NPV metrics for the chosen threshold. The main advantage of the proposed approach is that it provides a calibrated, interpretable, multivariable risk estimate derived from routine clinical data. Unlike fixed-rule indices, the model can capture non-linear effects and interactions, while interpretability tools (SHAP/permutation importance) and clinical–utility evaluation (threshold analysis and DCA) support transparent, clinically actionable deployment.

Overall, the results confirm that machine learning models, especially Random Forest, are able to effectively detect advanced fibrosis (F3–F4) from routine clinical laboratory data, and key predictors are consistent with known pathophysiological mechanisms of chronic liver disease progression. The model demonstrates promising internal performance within our study population; however, its applicability to other clinical settings and broader patient populations remains uncertain until it is externally validated in larger, multicenter cohorts.

### 4.3. Future Directions and Implications

The results of this study highlight several implications for clinical practice and future research. In clinical settings, earlier recognition of AIH may be improved through targeted educational programs for primary care physicians and public awareness efforts focused on patients, particularly middle-aged women, presenting with unexplained liver enzyme elevation even in the absence of marked symptoms. Our findings also suggest that machine learning approaches, particularly the RandomForest model, may be useful for identifying advanced fibrosis (F3–F4) using routine clinical laboratory parameters, thereby supporting risk stratification in chronic liver disease. In addition, the development of national clinical guidelines or diagnostic algorithms for patients with chronic hepatitis of unknown etiology may help standardize and accelerate diagnostic pathways, including timely serological testing and liver biopsy or non-invasive fibrosis assessment. From a research perspective, the establishment of a prospective AIH registry in Central Asia would be an important next step to enable the systematic evaluation of case detection, treatment response, and long-term outcomes.

### 4.4. Study Limitations

This study has several limitations that should be considered when interpreting the results. Its retrospective and descriptive design carries risks of selection bias and incomplete data. Most patients were seen at a single, large, tertiary care center in Almaty, and despite referrals from other regions, the sample may not reflect patients with milder forms of AIH who did not reach specialized care.

Limitations of the machine learning study include sample size and potential patient heterogeneity, which may impact the robustness of fold estimates. Furthermore, some variables are represented by categorical ranges, which may reduce the informational precision compared to continuous values. Finally, nested cross-validation ensures internal validity; however, for clinical translation, external validation on an independent cohort and an analysis of the model’s transferability across centers and populations are necessary.

Despite these limitations, this study provides valuable real-world clinical data on AIH in a poorly studied region. While these findings require confirmation and expansion in future prospective studies, they already highlight the relevance of late diagnosis and its association with severe AIH in Kazakhstan.

## 5. Conclusions

To our knowledge, this is the first study describing the characteristics of autoimmune hepatitis in a Kazakhstani population (Central Asia). This study shows that AIH in Kazakhstan is diagnosed predominantly in the late stages, as 69% of patients already had advanced fibrosis or cirrhosis, and the median diagnostic delay is 24 months, which increases the risk of advanced fibrosis. The clinical profile of AIH in Kazakhstan is characterized by a high frequency of overlap syndromes—28.8% of patients had AIH-PBC, 6.3% had AIH-PSC, and 2.5% had triple overlap syndrome—as well as significant comorbidity—MASLD in 22.9% and extrahepatic autoimmune diseases in 25.4% of patients. This combination of cholestatic, metabolic, and autoimmune features forms an atypical clinical picture and complicates the timely recognition of AIH. The use of machine learning methods has shown that a combination of routine clinical and laboratory parameters allows for the accurate detection of the risk of developing advanced liver fibrosis (F3–F4).

Further work should be aimed at studying the reasons for the diagnostic delay in AIH and the effectiveness of specific therapy, as well as conducting external validation of the machine learning model for predicting the risk of developing liver cirrhosis in an independent cohort and analyzing the transferability of the model between centers and populations.

## Figures and Tables

**Table 1:** Baseline demographic and clinical characteristics of patients with AIH (*n* = 240).

Characteristic	N (%)
Age, years	49.3 ± 14.3 (median 51; IQR 39–61)
Female sex	209 (87.1%)
BMI, kg/m^2^	25.1 ± 4.5 (median 24.8; IQR 21.9–27.9)
Overweight/obesity ^†^	117 (48.8%)
Disease duration at diagnosis	
Duration, months	36.3 ± 44.8 (median 24; IQR 12–48)
≤12 months	98 (40.8%)
13–24 months	42 (17.5%)
>24 months	100 (41.7%)
Presenting symptoms	
Jaundice	52 (21.7%)
Pruritus	87 (36.2%)
Right upper quadrant pain	71 (29.6%)
Weakness/fatigue	154 (64.2%)
Weight loss	13 (5.4%)

^†^, defined as “overweight” or any degree of obesity in the obesity category.

**Table 2:** Immunologic, histologic, and disease stage characteristics at diagnosis (*n* = 240).

Variable	N (%)
ANA positive	206 (85.8%)
ASMA positive	14 (5.8%)
AMA positive (any)	110 (45.8%)
Anti-mitochondrial M2 (AMA-M2) positive	42 (17.5%)
Elevated IgG	184 (76.7%)
Elevated IgM	65 (27.1%)
Elevated γ-globulins	171 (71.2%)

**Table 3:** Logistic regression analysis of factors associated with diagnostic delay >12 months.

Variable	β (Coefficient)	SE	OR	95% CI	*p*-Value
Advanced fibrosis/cirrhosis (≥F3)	0.7232	0.2997	2.06	1.15–3.71	0.0158
Age (years)	−0.0071	0.0098	0.99	0.97–1.01	0.4719
Female sex	−0.5054	0.4170	0.60	0.27–1.37	0.2255
Constant	0.6697	0.5851	—	—	0.2524

SE, standard error; OR, odds ratio; CI, confidence interval.

**Table 4:** Threshold-dependent classification metrics (outer test folds).

Operating Point	Threshold (Mean ± SD)	Sensitivity	Specificity	PPV	NPV
Fixed 0.5	0.500 ± 0.000	0.848 ± 0.045	0.557 ± 0.184	0.789 ± 0.071	0.656 ± 0.040
Youden-optimal	0.591 ± 0.044	0.766 ± 0.153	0.701 ± 0.122	0.834 ± 0.045	0.636 ± 0.105
Sensitivity ≥ 0.85	0.474 ± 0.030	0.867 ± 0.033	0.533 ± 0.165	0.782 ± 0.067	0.676 ± 0.025

## Data Availability

The data presented in this study are available from the corresponding author upon reasonable request due to the data protection restrictions of the clinic.

## Abbreviations

The following abbreviations are used in this manuscript:

AFP	Alpha-fetoprotein
AIH	Autoimmune hepatitis
ALP	Alkaline phosphatase
ALT	Alanine aminotransferase
AMA	Antimitochondrial antibodies
ANA	Antinuclear antibodies
Anti-HCV	Antibodies to the hepatitis C virus
Anti-M2	Anti-mitochondrial M2 antibodies
APRI	AST to Platelet Ratio Index
AST	Aspartate aminotransferase
BMI	Body mass index
COPD	Chronic obstructive pulmonary disease
CT	Computed tomography
Fib-4	Fibrosis-4 score
GGT	Gamma-glutamyl transferase
HBsAg	Hepatitis B surface antigen
HIV	Human immunodeficiency virus
IAIHG	International Autoimmune Hepatitis Group
IgG	Immunoglobulin G
IgM	Immunoglobulin M
IQR	Interquartile range
IST	Immunosuppressive therapy
kPa	Kilopascal
LKM	Liver kidney microsomal antibodies
MASLD	Metabolic dysfunction-associated fatty liver disease
p-ANCA	Perinuclear anti-neutrophil cytoplasmic antibodies
PBC	Primary biliary cholangitis
PSC	Primary sclerosing cholangitis
SD	Standard deviation
ASMA/SMA	Anti–smooth muscle antibodies
TE	Transient elastography
UDCA	Ursodeoxycholic acid
ULN	Upper limit of normal
